# Effects of cold-acclimation on gene expression in Fall field cricket *(Gryllus pennsylvanicus)* ionoregulatory tissues

**DOI:** 10.1186/s12864-017-3711-9

**Published:** 2017-05-08

**Authors:** Lauren E. Des Marteaux, Alexander H McKinnon, Hiroko Udaka, Jantina Toxopeus, Brent J. Sinclair

**Affiliations:** 1 Department of Biology, The University of Western Ontario, London, ON Canada; 2 Present Address: Faculty of Medicine, University of Manitoba, Winnipeg, MB Canada; 3 Present Address: Graduate School of Science, Biological Sciences, Kyoto University, Kyoto, Japan

**Keywords:** Insect, Cold tolerance, RNA-Seq, Acclimation, Ion pump, Malpighian tubules, Rectum, Phenotypic plasticity, *Gryllus*

## Abstract

**Background:**

Cold tolerance is a key determinant of temperate insect distribution and performance. Chill-susceptible insects lose ion and water homeostasis during cold exposure, but prior cold acclimation improves both cold tolerance and defense of homeostasis. The mechanisms underlying these processes are mostly unknown; cold acclimation is thought to enhance ion transport in the cold and/or prevent leak of water and ions. To identify candidate mechanisms of cold tolerance plasticity we generated transcriptomes of ionoregulatory tissues (hindgut and Malpighian tubules) from *Gryllus pennsylvanicus* crickets and compared gene expression in warm- and cold-acclimated individuals.

**Results:**

We assembled a *G. pennsylvanicus* transcriptome *de novo* from 286 million 50-bp reads, yielding 70,037 contigs (~44% of which had putative BLAST identities). We compared the transcriptomes of warm- and cold-acclimated hindguts and Malpighian tubules. Cold acclimation led to a ≥ 2-fold change in the expression of 1493 hindgut genes (733 downregulated, 760 upregulated) and 2008 Malpighian tubule genes (1009 downregulated, 999 upregulated). Cold-acclimated crickets had altered expression of genes putatively associated with ion and water balance, including: a downregulation of V-ATPase and carbonic anhydrase in the Malpighian tubules and an upregulation of Na^+^-K^+^ ATPase in the hindgut. We also observed acclimation-related shifts in the expression of cytoskeletal genes in the hindgut, including actin and actin-anchoring/stabilizing proteins, tubulin, α-actinin, and genes involved in adherens junctions organization. In both tissues, cold acclimation led to differential expression of genes encoding cytochrome P450s, glutathione-S-transferases, apoptosis factors, DNA repair, and heat shock proteins.

**Conclusions:**

This is the first *G. pennsylvanicus* transcriptome, and our tissue-specific approach yielded new candidate mechanisms of cold tolerance plasticity. Cold acclimation may reduce loss of hemolymph volume in the cold by 1) decreasing primary urine production via reduced expression of carbonic anhydrase and V-ATPase in the Malpighian tubules and 2) by increasing Na^+^ (and therefore water) reabsorption across the hindgut via increase in Na^+^-K^+^ ATPase expression. Cold acclimation may reduce chilling injury by remodeling and stabilizing the hindgut epithelial cytoskeleton and cell-to-cell junctions, and by increasing the expression of genes involved in DNA repair, detoxification, and protein chaperones.

**Electronic supplementary material:**

The online version of this article (doi:10.1186/s12864-017-3711-9) contains supplementary material, which is available to authorized users.

## Background

Most insects are chill-susceptible, such that their thermal performance and survival are limited in the cold at temperatures well above the freezing point [1]. Although ice formation causes direct injury, the mechanisms of chilling injury (i.e those not associated with ice formation) are not well-understood. Direct cold shock probably causes immediate damage to cells, for example via phase transition in membranes [2–4], disruption of the cytoskeleton [4–8], or induction of apoptosis [9]. Indirect chilling injury accumulates over time, most likely resulting from a loss of ion and water balance in the cold [10–14] (although there is also evidence of roles for oxidative damage and disruption of signalling pathways [5, 15, 16]). Cold-acclimated insects maintain water and ion homeostasis and avoid chilling injury and mortality to lower temperatures than warm-acclimated conspecifics [17–22], although the underlying mechanisms are not completely understood.

Chill-susceptible insects lose hemolymph water and Na^+^ to the gut lumen during cold exposure [13, 23–25] and re-establish this homeostasis during recovery upon rewarming [14]. Ion and water homeostasis in insects is primarily regulated at the Malpighian tubules and hindgut [26]. The distal Malpighian tubules actively transport ions across a leaky epithelium to drive secretion of water, metabolic wastes, and other ions into the tubule lumen. This primary urine – which is isosmotic to the hemolymph [27] – is partially modified at the proximal tubule (a tight epithelium) prior to entering the gut lumen [28–30]. Water and ions are then selectively reabsorbed from the gut lumen by the hindgut (particularly at the rectum) [31]. Na^+^-K^+^ ATPase (NKA) maintains high paracellular [Na^+^] in the rectal epithelium, driving paracellular migration of water (and concurrent reabsorption of some Na^+^ and Cl^-^) from the gut lumen to the hemolymph. Secretion and reabsorption are regulated by a suite of diuretic and antidiuretic peptides (see [32]), and these peptides may be important for recovery from cold stress in *Drosophila* [33]. Loss and recovery of ion and water balance in the cold is likely dependent upon processes at the Malpighian tubule and hindgut epithelia; specifically, enhanced ion pumping rate at low temperatures could maintain ion balance for longer, and decreased epithelial permeability could reduce the rate of ion leakage.

Transport enzyme function is temperature-dependent [34], thus cold exposure should limit ion pumping rates while the rate of passive leak should remain relatively unchanged [35]. To compensate, insects could increase transport capacity by expressing or mobilizing more transport enzymes [2, 36–38]. An obvious hypothesis, then, is that expression or activity of NKA and other transport enzymes in the Malpighian tubules and hindgut may be targeted for modification during cold acclimation. Water and ion leak across the ionoregulatory epithelia during cold exposure will depend on tissue permeability [13, 35, 39], and paracellular pathways may be especially important as these shunts are the primary pathway for water and anion movement across both Malpighian tubule and rectal epithelia [28, 31, 40, 41]. Because paracellular shunts are plastic and may be temperature-sensitive [42–45], they are likely targets for modification during cold acclimation to reduce epithelial permeability.

Surprisingly few genes have been directly associated with cold tolerance in insects [2, 46], but transcriptomics approaches have revealed many candidates. For example, modified expression of cuticular genes appears to underlie cold tolerance variation among New Zealand stick insects [47, 48], and the gene *Frost* has been associated with recovery from cold exposure in *Drosophila* [49–53], although its function remains elusive [54]. Cold shock recovery in *Sarcophaga bullata* flesh flies changes the expression of genes related to the membrane and cytoskeletal structure, apoptosis, protein folding, oxidative stress, and signaling [55], and many of these genes (in addition to those involving autophagy and ion transport) are also modified with cold acclimation and rapid cold-hardening in *D. melanogaster* [56]. Most transcriptomic studies have explored responses to acute cold exposure (e.g. [52, 57]), or compared natural variation among populations and species whose underlying population differences may render the specific differences that drive cold-related phenotypes difficult to detect (e.g. [47]). Acclimation of a single population is therefore a useful approach to identify candidate mechanisms associated specifically with plasticity of cold tolerance.

A few single - population transcriptomic studies have identified transcriptomic changes associated with cold acclimation, e.g. MacMillan et al. [58] and Gerken et al. [56] recently identified key pathways and 1000s of genes associated with cold acclimation in *Drosophila melanogaster.* In these *Drosophila* datasets, modification of ion transport (particularly differential expression of Na^+^ transporters [58]) and cellular adhesion is consistent with our expectation that modulating epithelial transport is associated with cold acclimation. However, these and other related studies have examined the transcriptomes of either entire animals (e.g. [56, 58]), or mixed tissues (e.g. the entire excised heads of stick insects [47, 48]). Because the Malpighian tubules and hindgut effectively work antagonistically in insect ion and water balance, transcriptomic shifts in these epithelia are likely to be masked in whole-animal homogenates. Thus, a tissue-specific approach to transcriptomics is urgently needed to more precisely determine the cellular- and tissue-level changes underlying cold acclimation in chill-susceptible insects.

The fall field cricket, *Gryllus pennsylvanicus* (Orthoptera: Gryllidae) is a generalist omnivore of grassy habitats across the Eastern North American temperate zone [59]. The species is univoltine and overwinters in diapause in the soil as an egg [60]. Adult *G. pennsylvanicus* are chill-susceptible: they develop chilling injuries in as little as 12 h at 0 °C, and are killed by 3–5 d at this temperature [13]. *Gryllus pennsylvanicus* exhibits plasticity in cold tolerance, and has emerged as a model for understanding cold-induced loss of ion and water balance [13, 39, 61]. Briefly, when these crickets are exposed to cold, Na^+^, Ca^2+^, Mg^2+^, and water migrate from the hemolymph to the gut, hemolymph [K^+^] rises, and muscle equilibrium potential is lost [13, 39, 61]. Ion and water balance are actively re-established during recovery from cold [14]. Cold-acclimated *G. pennsylvanicus* have improved defense of ion and water homeostasis in the cold, a lowered critical thermal minimum, faster chill coma recovery time, and suffer lower rates of injury and mortality following cold shock [39]. Thus, the *G. pennsylvanicus* system is well-suited for exploring mechanisms of cold tolerance plasticity.

Here we took a tissue-specific comparative gene expression approach to understand the processes of cold acclimation in the transporting epithelia of chill-susceptible insects. We assembled a transcriptome for *G. pennsylvanicus* and compared the expression of Malpighian tubule and hindgut genes between warm- and cold-acclimated adults (with a focus on genes involved in ion and water homeostasis and cellular and junctional integrity). We aimed to generate mechanistic hypotheses about specific molecular underpinnings of cold acclimation, and provide insights into the causes of water and ion disruption during cold exposure.

## Methods

### Insect rearing

Our colony of *G. pennsylvanicus* originated from individuals collected in 2004 from the University of Toronto at Mississauga campus, Ontario and was reared under constant summer-like conditions (25 °C, 14 L:10 D photoperiod, 70% RH), as described previously [13, 39]. At approximately 8 weeks post-hatch, and prior to sexual maturation, female crickets were separated from males to prevent mating. Adult females at approximately 11 weeks post-hatch were used for all experiments.

### Acclimation and dissection

Crickets were first isolated individually in common summer-like conditions (25 °C, 14 L:10D photoperiod) in mesh-covered 177 mL transparent cups (Polar Plastics, Summit Food Distributors, London, ON, Canada) containing egg carton shelters, rabbit food, and water. Isolation prevented cannibalism and lasted 1 week. We then haphazardly assigned crickets into cold- and warm-acclimations (*n* = 30 per treatment). For warm acclimation, crickets remained in the rearing growth chamber under constant summer-like conditions. We cold-acclimated crickets in a Sanyo MIR 154 incubator (Sanyo Scientific, Bensenville, Illinois) at 10 L:14 D with temperature decreasing from 25 to 12 °C over 7 days followed by constant 12 °C for 3 weeks. This regime lowers the critical thermal minimum c. 2 °C, reduces chill coma recovery time by c. 65%, increases survival following 5 d at 0 °C by nearly 80%, and enhances maintenance of ion and water homeostasis in the cold [39]. While the two acclimation temperatures are likely to affect physiological ageing, the 4 weeks of acclimation represent approximately 20% of the adult cricket lifespan. Therefore we assume that the effect of physiological age on gene expression will be less than the effect of acclimation temperature [62].

Cricket hindguts were dissected as described previously [61] from live crickets immediately following the 4 weeks of warm- or cold-acclimation. Under Ringer’s solution in a Petri dish the hindgut (rectum, colon, and ileum) was cut away from the gastrointestinal tract and flushed of fecal material with approximately 3 mL of Ringer’s using a syringe (this procedure took < 3 min). Malpighian tubules were removed as a single bunch by detaching the ureter with forceps, rinsing briefly in Ringer’s, and blotting on a tissue. Malpighian tubules and hindguts were flash-frozen in liquid nitrogen. Three biological replicates for sequencing of hindgut and Malpighian tubule transcriptomes were each comprised of pooled tissues from ten individuals. Following dissection, crickets were killed by placing them in a freezer at -20 °C. To maximize transcript representation for the *de novo* assembly, warm- and cold-acclimated whole male and female adult crickets, eggs, and warm-acclimated juveniles were pooled and added to an additional 1.5 mL microcentrifuge tube and flash frozen in liquid nitrogen. All samples were stored at -80 °C until RNA extraction.

### RNA extraction & cDNA library preparation

We homogenized thawed tissues with a plastic micropestle (ThermoFisher Scientific, Ottawa ON, Canada) in 1.1 mL TRIzol (Invitrogen, Burlington ON, Canada), and extracted RNA according to manufacturer’s instructions. We purified RNA extracts using the RNeasy Mini kit (Qiagen, Mississauga ON, Canada) according to manufacturer’s instructions, measured absorbance at 260 nm to determine RNA concentrations, and checked for quality with an Agilent Bioanalyzer. cDNA library production and sequencing were performed by the Donnelly Sequencing Center (Toronto ON, Canada). At 13 samples per lane, each cDNA library was sequenced twice using the Illumina HiSeq2500 platform (Illumina, San Diego, CA) with single-end, 50-bp reads.

### De novo transcriptome assembly and annotation

We removed Illumina adapter sequences and discarded sequences shorter than 15 nucleotides or containing unknown bases using the Galaxy web service [63]. Sequenced libraries were then grouped and assembled *de novo* using Trinity release 2012-10-25 [64, 65] on the SHARCNET computing cluster (https://www.sharcnet.ca), with 1 GB Jellyfish Memory and a minimum contig length criterion of 100 nucleotides. We analyzed transcriptome assembly “completeness” compared to a database of arthropod Benchmark Universal Single Copy Orthologs (BUSCO) using BUSCO v1.22 [66]. We compared the contigs from the Trinity assembly to the National Centre for Biotechnology Information (NCBI) non-redundant (nr) protein database (September 2013) by BLASTx (e-value threshold = 1 × 10^-3^). Gene Ontology (GO) annotation (e-value threshold = 1 × 10^-6^) was based on SwissProt BLAST matches using Blast2GO version 2.7.2 [67]. To filter out transcriptional artifacts, misassembled transcripts, and poorly supported transcripts, we mapped the original cleaned sequence reads back onto the Trinity-assembly using Bowtie2 version 2.1.0 [68, 69] and reassembled them with the Cufflinks package version 2.1.1 [70]. We used Blast2GO [67] and the NCBI database to obtain putative identities and GO annotation for mapped transcripts. We accepted one hit for each transcript at an e-value threshold of 1 × 10^-3^.

### Gene expression analyses

We used normalized read counts of genes in warm- and cold-acclimated hindgut and Malpighian tubule libraries for differential gene expression analyses using the edgeR Bioconductor package [71] in R (v3.2.2, R Development Core Team, 2015 [72]). Because each biological replicate was sequenced twice (two technical sequencing replicates), read counts from these technical replicate libraries were summed for each gene. For analyses we retained only those genes with at least 10 counts per million in three of the six libraries being compared (warm- vs cold-acclimated hindguts each had three biological replicates) [71]. Filtering yielded 11,140 and 11,066 contigs for differential gene expression analyses of hindgut and Malpighian tubules, respectively. We compared gene expression profiles within tissues (i.e. warm- vs cold-acclimated), and also compared the hindgut and Malpighian tubules for genes that were uniquely up- or downregulated between those tissues with cold acclimation. Individual genes were considered differentially expressed if the absolute fold change was ≥ 2 and if the *P*-value adjusted for false discovery rate (FDR) was < 0.05.

We used contigs that met our criteria for inclusion (fold change ≥ 2, FDR-adjusted *P*-value < 0.05) to identify the GO terms associated with the responses to cold acclimation in each tissue (note that we did not formally compare GO terms among tissues or treatments). Differentially-expressed pathways in warm- and cold-acclimated tissues were analyzed using the Kyoto Encyclopedia of Genes and Genomes (KEGG [73]). KEGG identities were assigned to contigs by the KEGG Automatic Annotation Server [74], and differential expression analyses of pathway components were performed using the Generally Applicable Gene-set Enrichment (GAGE) and Pathview Bioconductor packages [75, 76] in R. These packages identify coordinated differential expression in gene sets (pre-defined, functionally-related groups of genes) [76]. We accepted pathways as differentially-expressed if the FDR-adjusted *P*-value was < 0.1.

## Results

**Fig. 1:**
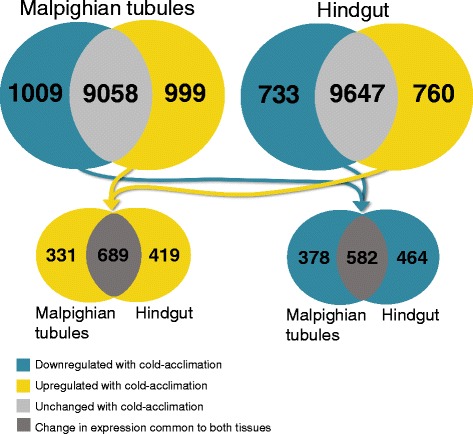
Number of genes upregulated, down-regulated, or unchanged in the hindgut and Malpighian tubules following cold acclimation. Differentially-expressed genes are those with an FDR alpha < 0.05 and a fold-change > 2. Note that due to some overlap in contigs the sum of genes up- or down-regulated across both tissues is less than the sum of genes up- or down-regulated in separate tissues (1439 and 1424 unique genes were up- and downregulated across both tissues, respectively)

Sequencing of 26 libraries yielded 286 million 50-bp reads, which we assembled into 70,037 contigs (Additional file 1: Table S1). Our transcriptome included 1808 (67.6%) complete and 415 (15.5%) fragmented arthropod BUSCOs; which is similar to other recent arthropod transcriptome assemblies (e.g. [77, 78]), and comparable to, or better than, the transcriptomes referred to by Simão et al. [66]. Approximately 44% of these contigs in our transcriptome had putative identities by BLAST (Additional file 2: Spreadsheet S1), and of these approximately 36% aligned to genes of the termite *Zootermopsis nevadensis*. Cold acclimation was associated with a two-fold or greater change in 1493 genes in the hindgut and 2008 genes in the Malpighian tubules (Fig. 1). Within a given tissue, the number of genes up- and down-regulated with cold acclimation were approximately equal. Approximately 52% of all upregulated genes and 60% of all downregulated genes exhibited unique differential expression across the two tissues. Eighty-one genes that appear to be important for cold acclimation (those with a 10-fold or greater change in expression) were unidentifiable by BLAST (Additional file 2: Spreadsheet S1). These represented 22 upregulated and 11 downregulated genes in the hindgut, and 26 upregulated and 22 downregulated genes in the Malpighian tubules.

The GO domain profiles that changed with cold acclimation were similar across the two tissues; of all up- or downregulated GO terms, just over half related to ‘molecular function’ (i.e. transport, binding, enzyme and receptor activities), over one third related to ‘biological processes’ (series of molecular events with a defined beginning and end), and roughly one tenth were ‘cellular components’ (i.e. specific locations of subcellular structures and macromolecular complexes; Additional file 3: Spreadsheet S2). Within the molecular function GO domain, genes involved in nucleotide, protein, metal, and ion binding accounted for over 50% of the upregulated transcripts and approximately 30% of downregulated transcripts in both tissues. Most of the cellular components that were differentially-expressed with cold acclimation involved the cell membrane and extracellular region. In the Malpighian tubules, genes pertaining to the cytoskeleton and cellular junctions accounted for 5% of all upregulated cellular component transcripts, while V-ATPase accounted for 2% of the downregulated transcripts. Metabolic genes accounted for much of the downregulated biological process transcript counts in both tissues. However, some unique differences in biological process profiles between the two tissues were apparent; approximately 17% of downregulated transcripts in the Malpighian tubules were transport-related (2% of which were ion transport-specific), compared to just over 8% of downregulated transcripts in the hindgut. Of the upregulated biological processes over 30% of transcripts in the hindgut involved stress response, protein folding, and repair, while over 10% of transcripts in the Malpighian tubules related to transport.

### Hindgut

Cold-acclimated *G. pennsylvanicus* had altered expression of putative gene orthologs related to apoptosis, the cytoskeleton, detoxification and repair, ion transport and pH regulation, phosphorylation, protein folding, and signal transduction in the hindgut (Tables 1 and 2). A complete list of differentially-expressed genes in the hindgut is provided in the Additional file 2: Spreadsheet S1. Upregulated genes involved in water and ion balance included those encoding atrial natriuretic peptide-converting enzyme, NKA α-subunit, and a Ca^2+^ release-activated Ca^2+^ channel protein, while downregulated genes included those putatively encoding bumetanide-sensitive Na^+^-Cl^+^ channel (the Na^+^-K^+^-2Cl^-^ cotransporter, or NKCC), carbonic anhydrase (CA) isozymes 1 and 9, and a mitochondrial Na^+^-H^+^ exchanger (NHA). A gene encoding the putative microtubule-associated protein Jupiter increased during cold acclimation, while a putative enzyme involved in homeoviscous adaptation – *∆*9 desaturase 1 – was downregulated 3.9-fold. The genes most differentially-expressed in cold-acclimated crickets related to repair and oxidative damage included those encoding cytochrome P450 (26-fold increase) and a putative cytochrome P450 cyp44 (5.5-fold decrease), glutathione-S-transferase (6.8-fold decrease), and vitellogenin (40-fold decrease). Cold acclimation corresponded with altered expression of hindgut heat shock proteins (*hsp 70* and *hsp 90* were upregulated, while *hsp 67B* and *hsp β11* were downregulated), and altered expression of some apoptosis genes. Cold-acclimated crickets also exhibited upregulation of a number of protein kinases, phosphodiesterases, and adenylate cyclase in the hindgut. Circadian genes *per, clock*, and *nocturnin* were upregulated with cold acclimation, while *timeless* was downregulated.

**Table 1:** Selected genes upregulated in the hindgut following cold acclimation whose putative function in relation to cold tolerance is discussed in the text

Function	Description	Fold change	*P*-value	Species
*Apoptosis*	Caspase-6	4.0	1.8E^-31^	*Gs*
	Caspase-8	4.0	9.1E^-39^	*Zn*
*Circadian*	Clock	2.7	1.7E^-17^	*Gb*
	Nocturnin	2.4	1.8E^-16^	*Zn*
	Period	7.4	1.6E^-50^	*Gb*
*Cytoskeleton*	Microtubule-Associated Protein Jupiter	4.2	5.3E^-31^	*Zn*
*Diuresis*	Atrial Natriuretic Peptide-Converting Enzyme	3.5	3.7E^-15^	*Zn*
*Ion transport*	Ca^2+^ Release-Activated Ca^2+^ Channel Protein 1	2.3	4.3E^-17^	*Zn*
	Na^+^-K^+^ ATPase Alpha Subunit	2.8	2.7E^-21^	*Ll*
*Neurotransmission*	Na^+^- and Cl^-^-Dependent GABA Transporter Ine	2.1	8.8E^-10^	*Md*
*Phosphorylation*	Dual Specificity Tyrosine-Phosphorylation-Regulated Kinase 2	2.7	2.2E^-04^	*Zn*
	Serine Threonine-Protein Kinase Rio3	2.9	3.7E^-07^	*Zn*
	Serine Threonine-Protein Kinase Sik3-Like Isoform X3	2.9	8.7E^-16^	*Ap*
*Protein folding/chaperone*	Heat Shock Protein 90	3.0	1.7E^-28^	*Gf*
	Hsp70 Family Member	2.1	8.0E^-14^	*Lm*
*Repair/detoxicant/antioxidant*	Cytochrome P450	26.0	7.7E^-108^	*Aa*
	Cytochrome P450 4C1	2.0	7.2E^-05^	*Zn*
	DNA Mismatch Repair Protein Mlh1	3.5	1.2E^-22^	*Xt*
	DNA Repair Protein Complementing Xp-G Cells	2.7	4.8E^-20^	*Zn*
	Glutathione S-Transferase D7	2.1	2.6E^-05^	*Zn*
*Signal transduction*	cAMP-Specific 3’,5’-Cyclic Phosphodiesterase, Isoform F Isoform *X*2	2.1	3.3E^-12^	*Tc*
	Dual 3’,5’ Cyclic-AMP and -GMP Phosphodiesterase 11	2.5	1.5E^-17^	*Zn*
	G Kinase-Anchoring Protein 1	3.8	6.5E^-34^	*Zn*
	G-Protein Coupled Receptor Mth2-Like	2.2	4.5E^-14^	*Ap*
	Protein Kinase C Iota (partial)	2.4	1.5E^-16^	*Zn*
*Signalling/gut contraction*	Adenylate Cyclase Type (partial)	2.5	1.2E^-13^	*Zn*

For a full list of the 760 upregulated hindgut genes, see Additional file 2: Spreadsheet S1. *P*-values were adjusted for false discovery rate (FDR). For each gene, the species with the highest sequence similarity via BLAST is given. Species codes: *Aa (Aedes aegypti), Ap (Acyrthosiphon pisum), Gb (Gryllus bimaculatus), Gf (Gryllus firmus), Gm (Galleria mellonella), Ll (Lutzomyia longipalpis), Lm (Locusta migratoria), Md (Microplitis demolitor), Ms (Modicogryllus siamensis), Tc (Tribolium castaneum), Xt (Xenopus tropicalis), Zn (Zootermopsis nevadensis)*

**Table 2:** Selected genes downregulated in the hindgut following cold acclimation whose putative function in relation to cold tolerance is discussed in the text

Function	Description	Fold change	*P*-value	Species
*Apoptosis*	Apoptosis-Inducing Factor 3-Like	-4.4	6.1E^-29^	*Nv*
*Circadian*	Timeless	-4.3	2.3E^-36^	*Gb*
*Ion transport*	Bumetanide-Sensitive Na^+^-Cl^-^ (partial) (NKCC)	-2.9	6.9E^-25^	*Zn*
	Na^+^-Independent Sulfate Anion Transporter-Like	-2.8	1.9E^-12^	*Mr*
	Organic Cation Transporter Protein	-2.6	4.2E^-19^	*Zn*
*Ion transport/pH regulation*	Carbonic Anhydrase 1	-4.6	4.9E^-49^	*Tc*
	Carbonic Anhydrase 9	-3.9	6.5E^-40^	*Zn*
	Mitochondrial Na^+^-H^+^ Exchanger NHA2	-2.9	7.0E^-18^	*Zn*
*Phospholipid biochemistry*	∆9 Desaturase 1	-3.9	1.4E^-10^	*Ad*
*Protein folding/chaperone*	Heat Shock Protein 67B2	-2.1	3.3E^-09^	*Zn*
	Heat Shock Protein β-11	-4.5	5.1E^-29^	*Zn*
*Repair/detoxicant/antioxidant*	Antioxidant Enzyme	-2.0	1.6E^-12^	*Go*
	Cytochrome P450 4C1	-4.1	4.0E^-42^	*Zn*
	Cytochrome P450 6A14	-3.8	1.3E^-12^	*Zn*
	Cytochrome P450 9E1	-2.5	9.5E^-19^	*Dp*
	Cytochrome P450 Cyp44	-5.5	1.9E^-43^	*Zn*
	Epsilon Glutathione S-Transferase	-2.1	7.8E^-11^	*Lm*
	Glutathione S-Transferase	-6.8	1.2E^-35^	*Bt*
	Glutathione S-Transferase-Like	-2.5	2.3E^-15^	*Md*
	Glutathione S-Transferase Sigma 1	-2.5	2.1E^-10^	*Sg*
	Glutathione S-Transferase Sigma 7	-4.8	1.5E^-26^	*Lm*
	Glutathione S-Transferase Theta 1	-2.8	2.5E^-21^	*Lm*
	Peroxiredoxin	-2.6	4.3E^-17^	*Go*
	Vitellogenin	-40.0	1.7E^-47^	*Ar*
	Vitellogenin-2	-18.4	1.2E^-50^	*Ps*

For a full list of the 733 downregulated hindgut genes, see Additional file 2: Spreadsheet S1. *P*-values were adjusted for false discovery rate (FDR). For each gene, the species with the highest sequence similarity via BLAST is given. Species codes: *Ac (Acheta domesticus), Ap (Aphis gossypii), Ar (Athalia rosae), Bt (Bemisia tabaci), Dp (Diploptera punctata), Gb (Gryllus bimaculatus), Go (Gryllus orientalis), Lm (Locusta migratoria), Md (Microplitis demolitor), Mr (Megachile rotundata), Nv (Nasonia vitripennis), Ps (Plautia stali), Sg (Schistocerca gregaria), Tc (Tribolium castaneum), Zn (Zootermopsis nevadensis)*

**Fig. 2:**
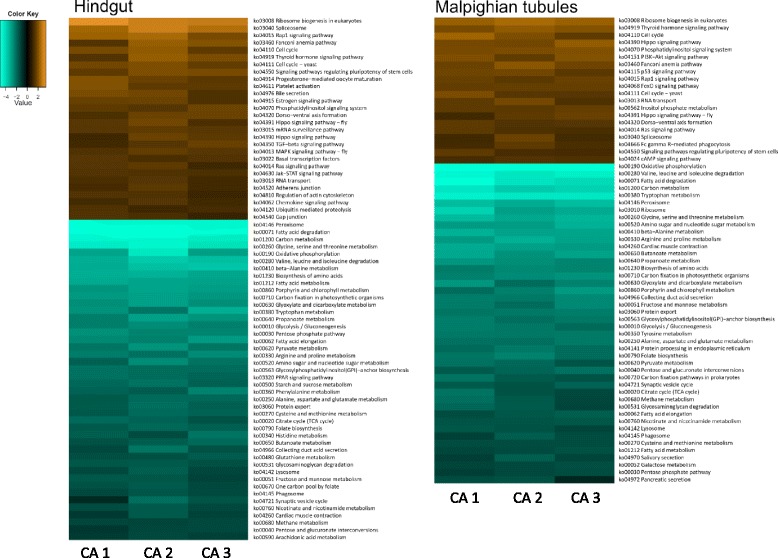
Heat map of differentially-expressed KEGG pathways in the hindgut and Malpighian tubules following cold acclimation. Upregulated pathways are given in orange and downregulated pathways are given in blue. Each heat map contains three column indicating three cold-acclimated biological replicates (CA 1-3) each compared to the mean expression among warm-acclimated replicates. For a complete description of each pathway, see the KEGG online resource (http://www.genome.jp/kegg/)

**Fig. 3:**
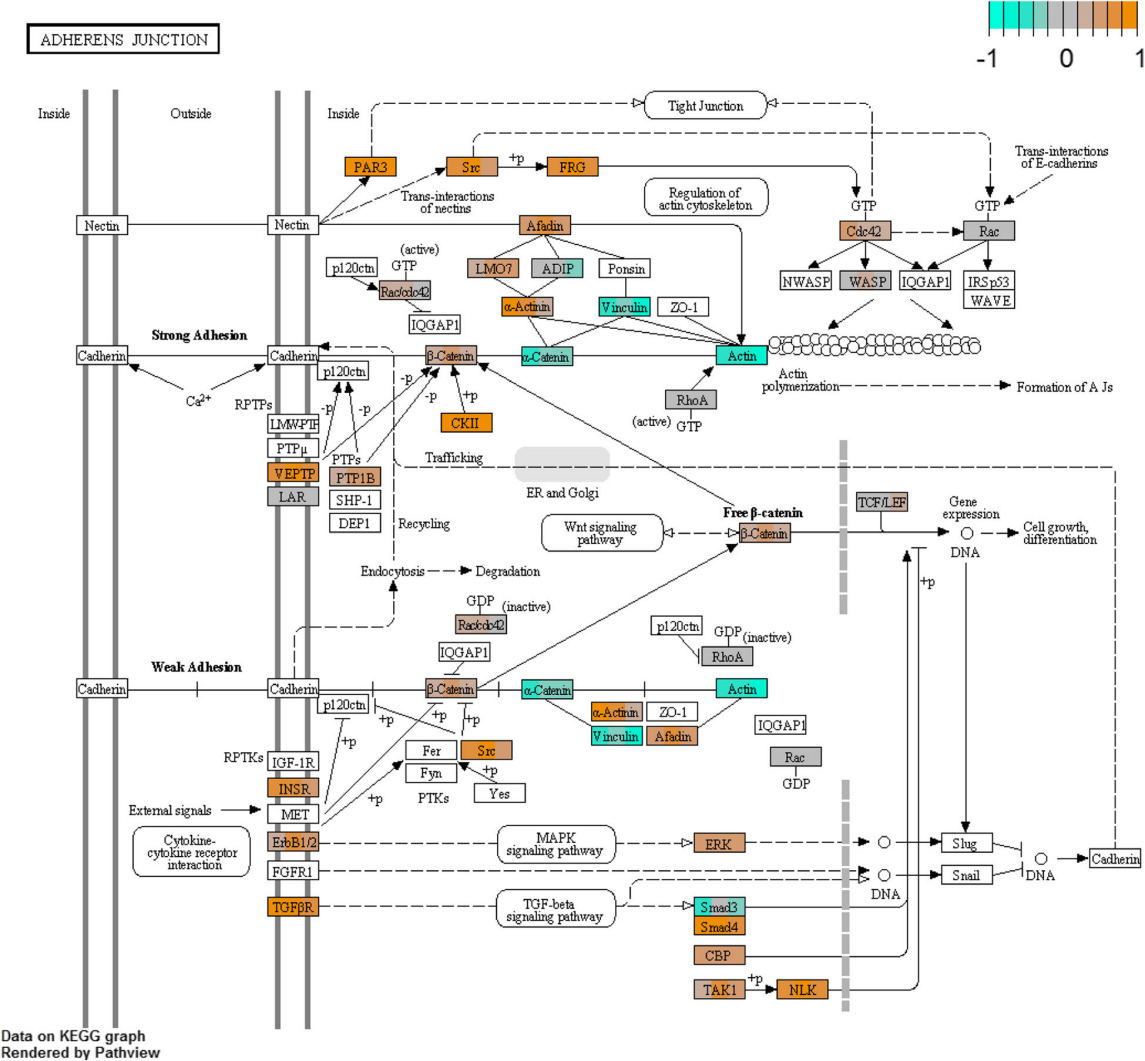
Shifts in the expression of ‘adherens junction’ KEGG pathway components in the cricket hindgut following cold acclimation, as an example of a pathway that was significantly differentially regulated. Each pathway component contains three color bars indicating three cold-acclimated biological replicates each compared to the mean expression among warm-acclimated replicates. For cold-acclimated crickets relative to warm-acclimated crickets, shifts in expression are either upregulated (*orange*), downregulated (*blue*), or unchanged (*grey*). For a complete description of each pathway component, see the KEGG ‘adherens junction’ reference pathway (http://www.genome.jp/kegg-bin/show_pathway?ko04520)

**Fig. 4:**
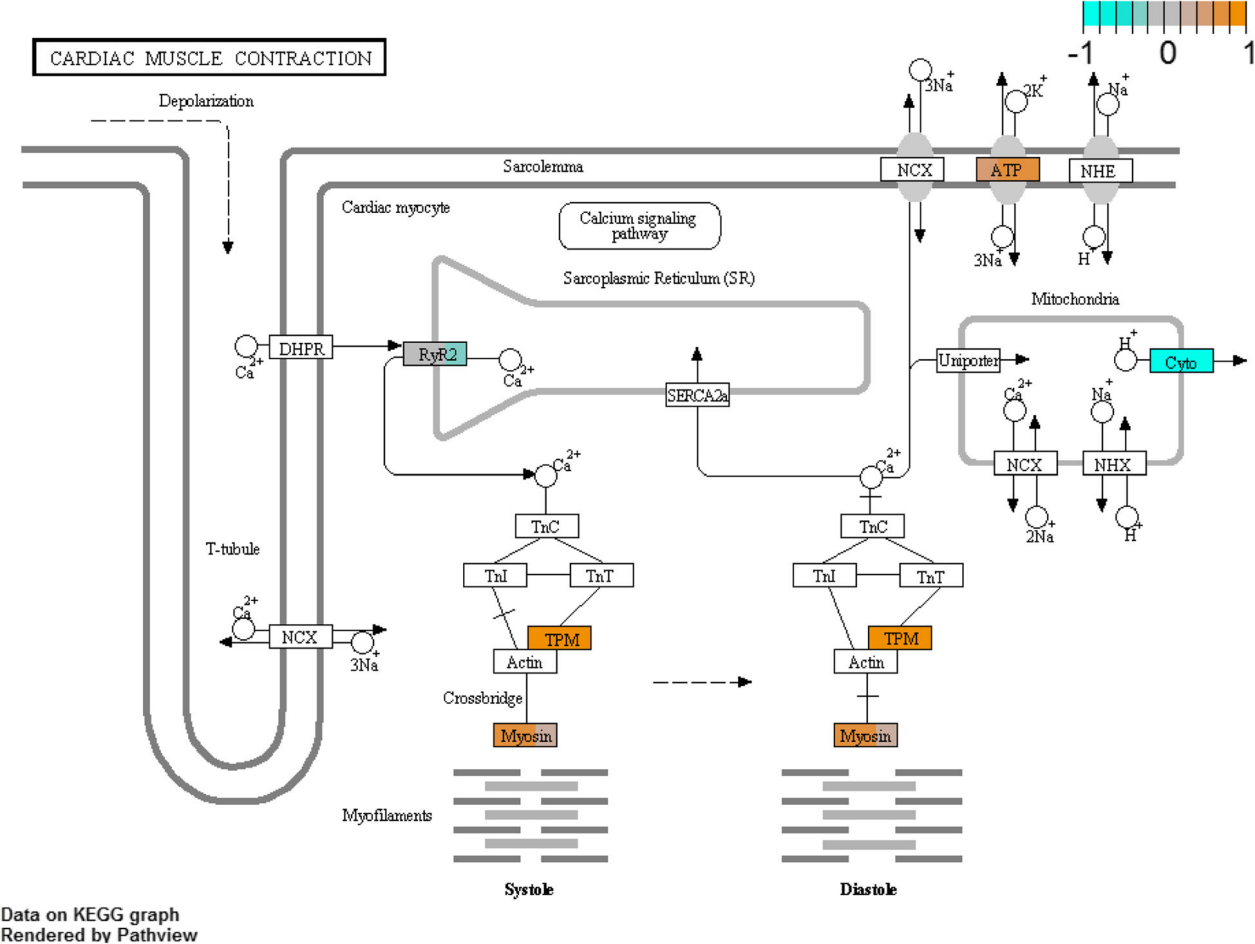
Shifts in expression of the ‘cardiac muscle contraction’ KEGG pathway components in the cricket hindgut following cold acclimation, as an example of a pathway that was significantly differentially regulated. Each pathway component contains three color bars indicating three cold-acclimated biological replicates each compared to the mean expression among warm-acclimated replicates. For cold-acclimated crickets relative to warm-acclimated crickets, shifts in expression are either upregulated (*orange*), downregulated (*blue*), or unchanged (*grey*). ATP - Na^+^-K^+^ ATPase α subunit, Cyto - cytochrome c reductase iron-sulfur subunit, TPM - tropomyosin 1, Myosin - myosin heavy chain 6/7. For a complete description of each pathway component, see the KEGG ‘cardiac muscle contraction’ reference pathway (http://www.genome.jp/kegg-bin/show_pathway?ko04260)

More KEGG pathways in the hindgut were downregulated with cold acclimation than were upregulated (Fig. 2). Among 25 upregulated pathways, ‘adherens junction’ (Fig. 3) and ‘gap junction’ are likely to be directly relevant to ion and water balance. Actin regulation within the ‘adherens junction’ pathway was modified; some genes putatively encoding actin-associated proteins (FRG and α-actinin) were upregulated while others were downregulated (β/γ actin, vinculin, and α-catenin). The putative proteins vascular endothelial protein tyrosine phosphatase (VE-PTP), transforming growth factor β2 (TGFβ2), and partitioning defective protein 3 (PAR3) were also upregulated. Upregulation of the ‘gap junction’ pathway was driven by increased expression of a gene putatively encoding tubulin α (TUBA), and to some degree epidermal growth factor receptor (EGFR, or ErbB-1,1 listed as the receptor tyrosine kinase, RTK), while the gene encoding protein kinase C α (PKC-α) was downregulated. Many of the 47 downregulated KEGG pathways in the hindgut were related to metabolism, but also included ‘cardiac muscle contraction’ (Fig. 4) and ‘synaptic vesicle cycle’. Downregulation of the ‘cardiac muscle contraction pathway’ was driven by a decrease in expression of the gene encoding cytochrome c reductase, however there were also significant increases in the expression of genes encoding the NKA α subunit, tropomyosin 1, and myosin heavy chain 6/7. Downregulation of the ‘synaptic vesicle cycle’ was driven by a reduction in the expression of the putative proton pump (V-ATPase), however the gene encoding the dynamin GTPase increased.

### Malpighian tubules

As in the hindgut, the Malpighian tubules of cold-acclimated crickets had altered expression of genes related to apoptosis and autophagy, the cytoskeleton, detoxification and repair, ion transport, pH regulation, phosphorylation and signal transduction, and protein folding (Tables 3 and 4). A complete list of differentially-expressed genes in the Malpighian tubules is provided in Additional file 2: Spreadsheet S1. Differentially-expressed genes involved in ion balance and pH regulation included a putative V-ATPase (downregulated 2-fold), Ca^2+^ and anion transporters and, as in the hindgut, a downregulation of both CA 1 and 9. Cold acclimation led to variable expression of multiple cytoskeletal genes, increased expression of two apoptosis genes, and decreased expression of one gene involved in autophagosome formation. Similar to the hindgut, cold-acclimated Malpighian tubules also exhibited increased expression of *hsp 70* and downregulation of *hsp 67B*, and both up- and downregulation of multiple repair and antioxidant genes (including those encoding putative cytochrome P450s and glutathione-S-transferases). Multiple kinase genes were upregulated in cold-acclimated Malpighian tubules (similar to the hindgut) in addition to a relatively large decrease (6.9-fold) in putative inositol monophosphatase expression. Altered expression of circadian genes following cold acclimation were also similar to that of the hindgut, and juvenile hormone expression was reduced nearly 11-fold.

**Table 3:** Selected upregulated genes in the Malpighian tubules following cold acclimation whose putative function in relation to cold tolerance is discussed in the text

Function	Description	Fold change	*P*-value	Species
*Apoptosis*	Caspase-8	2.1	1.7E^-5^	*Zn*
	Programmed Cell Death Protein 2	2.4	7.0E^-10^	*Nv*
*Circadian*	Clock	2.5	6.4E^-9^	*Gb*
	Nocturnin	3.5	1.2E^-23^	*Zn*
	Period	7.6	2.5E^-63^	*Ms*
*Cytoskeleton*	Gamma-Tubulin Complex Component 6	2.2	8.6E^-10^	*Zn*
	Kinesin-Like Protein Costa	2.8	2.6E^-9^	*Zn*
	Protein Shroom	2.5	1.6E^-9^	*Zn*
	Microtubule-Associated Protein Jupiter	2.2	1.8E^-8^	*Zn*
*Ion transport*	Ca^2+^-Transporting ATPase Type 2C Member 1	2.2	1.1E^-8^	*Zn*
	Solute Carrier Organic Anion Transporter Family Member 5a1	2.4	3.0E^-11^	*Hs*
*Phosphorylation*	Inositol Polyphosphate Multikinase	2.2	2.6E^-15^	*Zn*
	Serine Threonine-Protein Kinase rio3	3.4	5.4E^-35^	*Zn*
	Serine Threonine-Protein Kinase pctaire-2	2.6	3.9E^-14^	*Zn*
	Serine Threonine-Protein Kinase Tousled-Like 2	2.9	2.7E^-11^	*Zn*
	Tyrosine-Protein Kinase Transmembrane Receptor ror1	2.5	6.6E^-10^	*Phc*
	Tyrosine-Protein Phosphatase Non-Receptor Type 23	2.5	1.6E^-15^	*Zn*
*Protein folding/chaperone*	Hsp 70 Family Member	2.0	1.0E^-10^	*Lm*
	Hsp 70-binding Protein 1	3.3	2.7E^-22^	*Zn*
*Repair/detoxicant/antioxidant*	Alkylated DNA Repair Protein Alkb-Like Protein 4	2.6	1.4E^-12^	*Zn*
	Cytochrome P450	14.6	3.7E^-58^	*Aa*
	Cytochrome P450 2j2	3.4	4.6E^-7^	*Zn*
	Cytochrome P450 partial	6.1	9.1E^-11^	*Zn*
	DNA Mismatch Repair Protein mlh1	4.2	3.9E^-24^	*Xt*
	Glutathione S-Transferase Sigma 7	2.3	9.5E^-9^	*Zn*
	Peroxiredoxin-6	2.1	4.0E^-9^	*Lm*
	Probable Cytochrome P450 (partial)	6.2	2.6E^-13^	*Tc*
*Signal transduction*	cAMP-Specific 3’,5’-Cyclic Isoform F Isoform *X*2	3.2	2.4E^-30^	*Tc*
	G Kinase-Anchoring Protein 1	3.7	1.2E^-28^	*Zn*

For a full list of the 999 upregulated Malpighian tubule genes, see Additional file 2: Spreadsheet S1. *P*-values were adjusted for false discovery rate (FDR). For each gene, the species with the highest sequence similarity via BLAST is given. Species codes: *Aa (Aedes aegypti), Gb (Gryllus bimaculatus), Hs (Harpegnathos saltator), Lm (Locusta migratoria), Ms (Modicogryllus siamensis), Nv (Nasonia vitripennis), Phc (Pediculus humanus corporis), Tc (Tribolium castaneum), Xt (Xenopus tropicalis), Zn (Zootermopsis nevadensis)*

**Table 4:** Selected downregulated genes in the Malpighian tubules following cold acclimation whose putative function in relation to cold tolerance is discussed in the text

Function	Description	Fold change	*P*-value	Species
*Autophagy*	Autophagy-Related Protein 2-Like Protein B	-2.1	1.5E^-7^	*Zn*
*Circadian*	Timeless	-3.0	3.3E^-13^	*Gb*
*Cytoskeleton*	Microtubule-Associated Proteins 1A 1B Light Chain 3C	-4.9	8.5E^-23^	*Zn*
	Epidermal Growth Factor Receptor Kinase Substrate 8-Like Isoform X1	-2.4	1.9E^-9^	*Md*
	Gamma-Tubulin Complex Component 3	-2.0	4.7E^-9^	*Zn*
*Development*	Juvenile Hormone-Inducible	-10.6	3.8E^-50^	*Aa*
*Ion transport/pH regulation*	Carbonic Anhydrase 9	-4.2	2.1E^-33^	*Zn*
	Carbonic Anhydrase 1	-2.2	5.9E^-15^	*Tc*
	V-ATPase Subunit D	-2.1	1.2E^-14^	*Lm*
*Phosphorylation*	Inositol Monophosphatase	-6.9	1.1E^-58^	*Zn*
	Serine Threonine-Protein Phosphatase 2B Catalytic Subunit 2-Like Isoform *X*2	-2.1	9.0E^-14^	*Tc*
*Protein folding/chaperone*	Heat Shock Protein 67B2	-2.8	1.6E^-16^	*Zn*
*Repair/detoxicant/antioxidant*	Antioxidant Enzyme	-2.9	3.7E^-23^	*Go*
	Cytochrome P450	-2.9	1.4E^-11^	*Bt*
	Cytochrome P450 4C1	-2.7	3.2E^-22^	*Zn*
	Cytochrome P450 6A14	-2.5	3.2E^-13^	*Zn*
	Cytochrome P450 9E2	-2.3	1.4E^-11^	*Zn*
	Cytochrome P450 (partial)	-2.3	3.6E^-12^	*Zn*
	Cytochrome P450 9E1	-2.2	3.3E^-14^	*Dp*
	Glutathione S-Transferase	-3.2	1.8E^-23^	*Bt*
	Glutathione S-Transferase Theta 1	-3.1	1.9E^-28^	*Lm*

For a full list of the 1009 downregulated Malpighian tubule genes, see Additional file 2: Spreadsheet S1. *P*-values were adjusted for false discovery rate (FDR). For each gene, the species with the highest sequence similarity via BLAST is given. Species codes: *Aa (Aedes aegypti), Bt (Bemisia tabaci), Dp (Diplotera punctata), Gb (Gryllus bimaculatus), Go (Gryllotalpa orientalis), Lm (Locusta migratoria), Md (Microplitis demolitor), Tc (Tribolium castaneum), Zn (Zootermopsis nevadensis)*

Similar to patterns in the hindgut, more KEGG pathways were downregulated than were upregulated in cold-acclimated Malpighian tubules (Fig. 2). Many of the 20 upregulated pathways were involved in signaling, and most of the 47 downregulated pathways related to metabolism. The ‘cardiac muscle contraction’ pathway (appropriate to insect striated muscle, [79]) was downregulated based on reduced expression of a cytochrome c reductase gene. Unlike in the hindgut, NKA, tropomyosin, or myosin heavy chain components of this pathway were not upregulated in Malpighian tubules. The ‘synaptic vesicle cycle’ pathway exhibited downregulation overall (driven by downregulation of V-ATPase), however a number of genes involved in endocytosis and vesicle-membrane fusion were upregulated. These upregulated genes include those encoding putative N-ethylmaleimide-sensitive factor (NSF, an ATPase), dynamin, AP2 complex α (a protein associated with endocytosis of clathrin-coated vesicles), and syntaxin 1A (a protein involved in vesicle fusion for exocytosis).

## Discussion

Cold-acclimated *Gryllus pennsylvanicus* exhibited modified expression of a range of genes, the functions of which were broadly consistent with differentially-regulated genes associated with cold acclimation and rapid cold-hardening in *Drosophila* [56, 58, 80]. In crickets, genes involved in stress response, protein folding, and repair were prominent in cold acclimated hindguts, while cold acclimation in the Malpighian tubules was associated with shifts in transport-related genes. In both tissues, cold acclimation was accompanied by altered expression of genes encoding components of the membrane and extracellular space.

### Water balance

Only one gene with known function in insect water homeostasis – that encoding atrial natriuretic peptide-converting enzyme – was upregulated in the hindgut following cold acclimation. The mosquito homologs of this enzyme stimulate primary urine production by the Malpighian tubules by increasing secretion of Na^+^ and Cl^-^ [32, 81]; the role of this peptide in the insect hindgut is less certain, but increased Na^+^ and Cl^-^ transport could enhance water reabsorption [82] and help to defend hemolymph volume during cold exposure. Although some aquaporins have been associated with insect freeze tolerance [83–85], their role in cold acclimation among chill-susceptible insects is unknown, and none of the water-transporting insect aquaporins [86] were differentially-expressed in the hindgut or Malpighian tubules with cold acclimation.

### Ion transport

Cold acclimation corresponded with altered expression of putative NKA, NKCC, CA, NHA, and V-ATPase–encoding genes, which are typically enriched in insect transporting epithelia [87]. Most of these gene expression changes were observed in the hindgut. Although all of these transport enzymes contribute to primary urine production by the Malpighian tubules [88], cold-acclimated Malpighian tubules only exhibited downregulation of genes encoding V-ATPase and CAs 1 and 9.

CA catalyzes the hydration of CO_2_ to produce H^+^ + HCO_3_^-^, a source of protons for export by apical V-ATPase by the Malpighian tubules [87, 89, 90], which (in exchange for Na^+^ or K^+^ by NHA and K^+^-H^+^ antiporters [87, 91]) drives passive excretion of water and anions [28, 92]. Downregulation of CA9 (membrane-bound), CA1 (cytosolic), and V-ATPase in the Malpighian tubules during cold acclimation could therefore have an antidiuretic effect, perhaps defending hemolymph volume in the cold. Indeed, cold-acclimated *G. pennsylvanicus* Malpighian tubules produce primary urine more slowly (Des Marteaux, Khazraeenia, and Sinclair, unpublished observations).

Paracellular Na^+^ gradients across the rectal pads drive passive reabsorption of water against osmotic gradients [31, 93, 94]. Failure of NKA to maintain these Na^+^ gradients during cold exposure could account for leak of Na^+^, and consequently water, to the gut. We might therefore expect cold-acclimated crickets to compensate for slower enzyme pumping rates at low temperatures by increasing NKA protein abundance; indeed, expression of the α (catalytic) subunit of NKA increased nearly 3-fold in the cricket hindgut after cold acclimation. In *D. melanogaster*, cold acclimation decreases whole-body NKA activity [22], suggesting a decoupling of Na^+^ gradients across the gut rather than compensation for slowed NKA activity in the cold [22]. However, it is unclear how NKA activity changes in *D. melanogaster* ionoregulatory tissues specifically, so we cannot unequivocally suggest that Diptera and Orthoptera use different acclimation strategies with regards to Na^+^ balance. NKCC (which imports Na^+^_,_ K^+^, and Cl^-^ basally, energizing apical ion exchange [87, 95]) was downregulated in cold-acclimated cricket hindguts, but the role of NKCC in insect hindgut transport has received little attention, making it difficult to evaluate how NKCC regulation might modify homeostasis.

### Cell junctions and structure

Cold acclimation was associated with altered expression of cell and tissue structure-related genes (e.g. hindgut genes involved in cell growth, differentiation, and adhesion included endothelial growth factor [96–98]), which could indicate a modified rectal pad epithelium, and which we hypothesise would enhance cold tolerance by minimizing Na^+^ and water leak in the cold [13]. In addition to possible roles in providing protons for ion transport, CA9 may have roles in cellular adhesion [99], proliferation, and differentiation (at least in mammals [100]). Thus, CA9 downregulation in hindgut and Malpighian tubules during cold acclimation may affect epithelial transport by increasing cellular adhesion and epithelial tightness. Tissue-specific post-translational modification of CA9 is an important means of regulating CA9 activity [101], which would not be captured in a transcriptome comparison such as the present study.

Cold-acclimated crickets had altered expression of hindgut cellular adhesion components associated with both adherens and septate junctions, which comprise the bulk of paracellular connections in the rectal pad [31, 102–104], and are closely related [105]. Cold acclimation was also associated with differential expression of genes encoding actin-membrane anchors, which could influence cell junction characteristics or cell shape. We hypothesise that altered actin-membrane anchoring could reduce tension, shearing damage, or unwanted stretch-activation of membrane-bound ion channels [4, 106] when the gut is distended by water migration during cold exposure.

Some gap and tight junction components were also altered during cold acclimation. These will likely modify ion and water recycling between the cytoplasm and paracellular channels [107, 108], and selectivity of absorption [109, 110]. Upregulation of PAR3 [97] and downregulation of PKC-α [111] suggests increased tight junction formation and therefore increased tightness of the hindgut epithelium during acclimation; we suggest that changes in tight junction morphology in the rectal pads following acclimation could be confirmed by transmission electron microscopy or immunostaining [112, 113]. After cold acclimation, we also observed shifts in the expression of multiple Malpighian tubule genes involving the cytoskeleton and cell junctions (e.g. protein shroom) [114, 115]. Whether these structural changes affect ion and water balance requires some assessment of Malpighian tubule permeability following cold acclimation.

### Chilling injury

Cold-attributed oxidative stress, disruption of homeostasis and signaling, protein mis-folding, and loss of membrane and cytoskeletal integrity may all contribute to chilling injury and mortality in chill-susceptible insects [12, 15, 116–120]. Cold acclimation was associated with upregulation of putative apoptosis genes (e.g. those encoding the apoptosis initiator caspase 6 and the apoptosis effector caspase 8 [121]), as well as shifts in autophagy-related gene expression (e.g. upregulation of Ras and ubiquitin signaling KEGG pathways). We hypothesize that the ability to clear cold-damaged cells or cell components is likely increased in cold-acclimated crickets. Polymorphisms or shifts in the expression of genes associated with apoptosis and autophagy appear to be common to the rapid cold-hardening process [56], and response to dehydration [122, 123] in other insects.

The cytoskeleton depolymerises at low temperatures in fish, mammals, and insects [6, 7, 119, 124], and damage to the cytoskeleton could well be associated with chilling injury in insects. Water loss – which occurs during cold exposure in chill-susceptible species – appears to drive shifts in cytoskeletal gene expression in other insects [122]. Cold-acclimated crickets had altered expression of cytoskeletal branching and stabilizing genes, such as α-catenin, ARP2/3 (a nucleation site for actin polymerization [125]), and (in the hindgut) tropomyosin, microtubule protein Jupiter and MAP1A/B. Together, this suggests enhanced polymerisation and stabilization of actin and microtubules. Cytoskeleton-related genes were also upregulated in cold-acclimated *Culex pipiens* [8], *Delia antiqua* [7], alfalfa (*Medicago sativa*) [4], and *D. melanogaster* [58, 126]*.*

Cold exposure appears to cause oxidative stress [16, 118, 127], and cold acclimation is associated with increased activity or expression of antioxidants (e.g. glutathione-S-transferase, catalase, and superoxide dismutase) in a number of insects [16, 46, 58, 128–130]. We observed increased expression of some putative DNA repair, and glutathione-S-transferase genes in both the hindgut and Malpighian tubules after cold acclimation (and decreased expression in some of these genes, which we attribute to decreased reactive oxygen species production in the cold [16, 131]). Insects often upregulate antioxidant expression during rewarming [16, 46, 128], so it is possible that a larger suite of antioxidants may become relevant if expression is also modified after cold exposure.

Cold acclimation was associated with increased expression of *hsp 70* in both tissues (as well as *hsp 90* in the hindgut). Heat shock proteins have a range of cellular-protective roles including as protein chaperones, and could therefore protect against a many aspects of thermal stress and maintain cellular integrity (and therefore perhaps epithelial function) during and after cold exposure. In cold-acclimated *D. melanogaster, hsp 70* is one of the few upregulated genes that also increases in protein abundance [80]. Genes encoding several hsps were downregulated, including the less-characterized *hsp β11* (involved in the vertebrate heat stress response [132]) and *hsp 67B2* (*hsp 67Bb),* which may protect against heat and oxidative stress in *Drosophila* [133, 134].

### Other candidate genes

Membrane remodelling is likely an important aspect of cold acclimation [2, 135, 136], and a large proportion of differentially-expressed GO cellular components in the cold-acclimated Malpighian tubules and hindgut were membrane-associated. In the hindgut, a gene encoding ∆9 desaturase (which is involved in homeoviscous adaptation) was downregulated with cold acclimation. By contrast, cold acclimation in the flies *Belgica antarctica* and *Delia radicum* is associated with upregulation of ∆9 desaturase [36, 137, 138]. Changes in membrane composition and fluidity with cold acclimation have not been explored in *G. pennsylvanicus*.

Loss of signal transduction in the cold is a proposed mechanism of chill coma, and recent evidence suggests that paralysis during cold exposure results from direct inhibitory effects of low temperature on neuromuscular function [24, 25, 61, 139, 140]. The expression of some signal transduction and neurotransmission genes (e.g. cAMP, G-proteins, PKC, and a GABA transporter and Ca^2+^ channel) was altered with cold acclimation in crickets, however it is difficult to predict how these changes would enhance cold tolerance. Perhaps of greater interest were vesicle localization and fusion genes such as dynamin (which mediates membrane-vesicle fusion), NSF (a vesicle-fusing ATPase), AP2 (involved in vesicle endocytosis), and syntaxin 1A (which promotes vesicle-membrane docking), all of which were upregulated with cold acclimation. Vesicle-membrane localization is important for both neurotransmission and recruitment of ion transporters (the latter of which could directly affect ion homeostasis in the cold [141]), and is likely slowed in the cold.

Cold acclimation appears to affect the expression of some genes associated with circadian rhythm, storage and metabolism, development, and phosphorylation. Differential expression of circadian genes was likely a result of the cold (e.g. low temperatures decrease the expression of *timeless* and increase the expression of *per* in flesh flies [142]). The expression of hindgut genes encoding vitellogenin (a yolk protein precursor) was drastically lower with cold acclimation, and this also occurs in cold-acclimated *D. melanogaster* [80]; in this case, we suspect that vitellogenin mRNA came from traces of fat body remaining on the gut during dissections [143].

## Conclusions

**Fig. 5:**
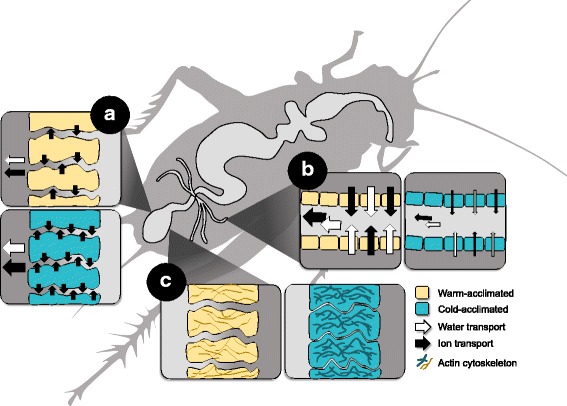
Candidate mechanisms of cold acclimation in *Gryllus pennsylvanicus*. **a** Increased expression of NKA in rectal pad epithelia should increase Na^+^ and water reabsorption; this may counteract leak of water and ions and aid in chill coma recovery). **b** Downregulation of CA and V-ATPase expression in the Malpighian tubules should slow primary urine production, thereby retaining hemolymph volume. **c** Cytoskeletal and junctional remodelling of the hindgut may mitigate water and ion leak during cold exposure

We have assembled the first transcriptome of *Gryllus pennsylvanicus* and our tissue-specific comparative approach yielded precise mechanistic hypotheses about the cold acclimation process. Cold acclimation appears to involve a modification of both ion transport function and cellular/junctional integrity (summarized in Fig. 5). The ion transport modifications likely defend hemolymph volume in the cold; decreased Malpighian tubule V-ATPase and CA expression should slow primary urine production, while upregulation of hindgut NKA should increase Na^+^ and water reabsorption. Remodeling of the cytoskeleton and adherens junctions (and potentially tight junctions) may mitigate paracellular leak of water and ions in both the hindgut and Malpighian tubules. Cold-acclimated crickets may prevent direct chilling injury by stabilizing the actin cytoskeleton and by changing the way in which actin anchors to the membrane. To repair chilling injuries, cold-acclimated insects may increase expression of antioxidant, DNA repair, apoptosis, autophagy, and chaperone genes. Upregulation of NKA (which should enhance Na^+^ and water reabsorption across the rectum) may also account for faster chill coma recovery in cold-acclimated insects.

## Additional files

Additional file 1: Table S1. Summary of *G. pennsylvanicus* transcriptome *de novo* assembly. (DOCX 12 kb)

Additional file 2: **Spreadsheet S1.** Complete list of genes differentially regulated in the hindgut and Malpighian tubules (MTs) following cold acclimation. Gene identities (Contig description) and GO term IDs were assigned by Blast2GO based on the top BLAST hit (GenBank accession number) of each contig. *P*-values were adjusted for false discovery rate (FDR). CPM, counts per million; FC, fold-change. (XLSX 356 kb)

Additional file 3: **Spreadsheet S2.** List of GO terms that were differentially represented in the hindgut and Malpighian tubules (MTs) following cold acclimation. Read counts of contigs associated with each GO term were summed for cold- and warm-acclimated crickets. Positive Δread counts (cold-acclimated cricket read counts minus warm-acclimated cricket read counts) indicate upregulation of a GO term in cold-acclimated crickets, while negative Δread counts indicate downregulation in cold-acclimated crickets. (XLSX 82 kb)

## Abbreviations

AP2: Adaptor-related protein complex 2 subunit α
ARP2/3: Actin-related proteins 2 and 3
bp: Base pair
CA1: Carbonic anhydrase isoform 1
CA9: Carbonic anhydrase isoform 9
CT_min_: Critical thermal minimum
EGFR/ErbB-1: Epidermal growth factor receptor
e-value: Expectation value
hsp: Heat shock protein
MAP1A/1B: Microtubule-associated proteins 1A/1B
NHA: Na^+^-H^+^ exchanger
NKA: Na^+^-K^+^ ATPase
NKCC: Bumetanide-sensitive Na^+^-K^+^-2Cl^-^ cotransporter
NSF: N-ethylmaleimide-sensitive factor
PAR3: Partitioning defective 3
PKC-i: Protein kinase C iota
PKC-α: Protein kinase C α
ROS: Reactive oxygen species
RTK: Receptor tyrosine kinase
TGFβ2: Transforming growth factor β2
TUBA: Tubulin α
V-ATPase: Vacuolar-type H^+^ ATPase
VE-PTP: Vascular endothelial protein tyrosine phosphatase
