# Supplementary material for: Effects of cold-acclimation on gene expression in Fall field cricket (Gryllus pennsylvanicus) ionoregulatory tissues
Source: BMC Genomics. 2017 May 8;18:357. doi: 10.1186/s12864-017-3711-9 (PMC5422886; doi:10.1186/s12864-017-3711-9)
Supplement: Supplementary file 1 — Summary of G. pennsylvanicus transcriptome de novo assembly. (DOCX 12 kb) [file 12864_2017_3711_MOESM1_ESM.docx]

**Supplementary material**

**Table S1**. Summary of *G. pennsylvanicus* transcriptome *de novo* assembly.

| **Sequencing & Quality Control** |  |
| --- | --- |
| Libraries | 26 |
| 50-bp reads (raw) | 286 million |
| 50-bp reads (trimmed/cleaned) | 266 million |
| **Trinity assembly** |  |
| Assembly length (bp) | 92 million |
| Contigs | 260,407 |
| Mean contig length (bp) | 352 |
| Median contig length (bp) | 156 |
| N50 | 716 |
| GC % | 38 |
| **Cufflinks refined assembly** |  |
| Length (bp) | 59 million |
| Contigs | 70,037 |
| Mean contig length (bp) | 839 |
| Median contig length (bp) | 459 |
| N50 | 1524 |
| GC % | 39 |
| **Identification** |  |
| Contigs with BLAST hit | 30,666 |
| Contigs with GO description | 5292 |
|  |  |
